# Prevalence and Demographic Correlates of Poor Sleep Quality Among Frontline Health Professionals in Liaoning Province, China During the COVID-19 Outbreak

**DOI:** 10.3389/fpsyt.2020.00520

**Published:** 2020-06-12

**Authors:** Yifang Zhou, Yuan Yang, Tieying Shi, Yanzhuo Song, Yuning Zhou, Zhibo Zhang, Yanan Guo, Xixi Li, Yongning Liu, Guojun Xu, Teris Cheung, Yu-Tao Xiang, Yanqing Tang

**Affiliations:** ^1^Department of Psychiatry, The First Affiliated Hospital of China Medical University, Shenyang, China; ^2^Department of Geriatric Medicine, The First Affiliated Hospital of China Medical University, Shenyang, China; ^3^Unit of Psychiatry, Faculty of Health Sciences, Institute of Translational Medicine, University of Macau, Macau, China; ^4^Center for Cognition and Brain Sciences, University of Macau, Macau, China; ^5^Department of Psychiatry, Southern Medical University Nanfang Hospital & Guangdong-Hong Kong-Macao Greater Bay Area Center for Brian Science and Brain-Inspired Intelligence, Guangzhou, China; ^6^Department of Nursing, The First Affiliated Hospital of Dalian Medical University, Dalian, China; ^7^School of Nursing, Hong Kong Polytechnic University, Hong Kong, Hong Kong

**Keywords:** COVID-19, China, health professionals, sleep quality, Pittsburgh Sleep Quality Index

## Abstract

**Background:**

Little empirical evidence is known about the sleep quality of frontline health professionals working in isolation units or hospitals during the novel coronavirus disease (COVID-19) outbreak in China. This study thus aimed to examine the prevalence of poor sleep quality and its demographic and correlates among frontline health professionals.

**Methods:**

This is a multicenter, cross-sectional survey conducted in Liaoning province, China. Sleep quality was measured by the Pittsburgh Sleep Quality Index (PSQI).

**Results:**

A total of 1,931 frontline health professionals were recruited. The prevalence of poor sleep quality was 18.4% (95%CI: 16.6%–20.11%). Multivariate logistic regression analysis found that older age (OR=1.043, 95%CI=1.026–1.061, *P* < 0.001), being nurse (OR=3.132, 95%CI=1.727–5.681, *P* < 0.001), and working in outer emergency medical team (OR=1.755, 95%CI=1.029–3.064, *P*=0.039) were positively associated with poor sleep quality. Participants who were familiar with crisis response knowledge were negatively associated with poor sleep quality (OR=0.70, 95%CI=0.516–0.949, *P*=0.021).

**Conclusion:**

The prevalence of poor sleep quality was relatively low among frontline health professionals during the COVID-19 epidemic. Considering the negative impact of poor sleep quality on health professionals’ health outcomes and patient outcomes, regularly screening and timely treatments are warranted to reduce the likelihood of poor sleep quality in health professionals.

## Introduction

The novel coronavirus disease (COVID-19) caused by the severe acute respiratory syndrome coronavirus 2 (SARS-CoV-2) has aroused enormous attention nationwide (1). This disease was first reported in Wuhan, Hubei province, and then was transmitted to other areas of China (2). As of 1st May, 2020, there have been 82,874 confirmed patients with COVID-19 and 4,633 deaths in China (3).

In order to control the rapid disease transmission, China has suspended public transport and adopted mass quarantine measures in multi-regions since late January, 2020 (2). The National Health Commission of China (NHC) has adopted a range of emergency measures, including the establishment of emergency isolation infectious units and hospitals, and temporary quarantine facilities (“Fang cang” hospitals) (4). External emergency medical teams have also been promptly established nationwide and assigned to provide medical assistance in Hubei province, China. As of 26^th^ February, 2020, more than 178 crisis response teams comprising 32,395 volunteer health professionals have been summoned to Hubei province (5).

Due to insufficient knowledge, awareness and preventive measures in the early stage of the COVID-19 outbreak, a total of 3,387 health professionals in 476 clinical sites have been infected with the disease, of which, 26 died (6). Frontline health professionals, especially those working in Hubei province, and having close contacts with infected patients often reported excessive workload. Due to insufficient supplies of full protective gear, dangerous working environments, and limited clinical experiences in managing the COVID-19, frontline health professionals are extremely vulnerable to experience fatigue, anxiety, depression, emotional breakdown, and sleep disturbance (7).

Sleep problems, such as poor sleep quality, are common in the health care profession due to high level of work-related stress (8, 9). Poor sleep quality could result in serious health consequences, such as hypertension, exhaustion, burnout, and depression (10–13). Health professionals suffering from poor sleep quality were more likely to have poor work performance, which could compromise patient safety and reduce the quality of patient care. In extreme case, health professionals could prescribe inaccurate diagnosis causing potentially fatal medical errors (14–19). Before developing preventive strategies and alleviating the negative outcomes of poor sleep quality, it is pivotal to understand its epidemiology and correlates among health professionals.

Sleep quality could be measured by both objective [e.g., polysomnography (PSG)] and subjective instruments [e.g., sleep diary, and Pittsburgh Sleep Quality Index (PSQI)] (20). The PSQI is the most commonly used subjective assessment tool measuring global sleep quality. A recent meta-analysis (21) showed that the pooled prevalence of poor sleep quality as measured by the PSQI was 61.0% in nurses.

Liaoning province is located in northern China. As of 1st May, 2020, there had been 146 COVID-19 patients in Liaoning province, of which, two died (3). During the COVID-19 outbreak, frontline health professionals experienced high work-related stress, which could lead to psychological distress, burnout, and sleep problems. To date, little has been known about the prevalence of sleep quality among frontline health professionals in areas of China which were less affected by the COVID-19. This gap gave us the impetus to examine the prevalence of poor sleep quality and its associated factors in this population. We hypothesized that frontline health professionals working in Liaoning province were less likely to experience poor sleep quality compared to their counterparts working in Hubei province—the epicenter of the COVID-19 outbreak.

## Methods

### Study Design and Participants

This was a cross-sectional study conducted between February 21 and 23, 2020 in Liaoning province, China using convenience snowball sampling. During the COVID-19 outbreak, frontline health professionals were managed by hospital authorities using the WeChat in Liaoning province, China. Data collection was executed using the Wenjuanxing program which is an application embedded with WeChat (https://www.wjx.cn/app/survey.aspx). WeChat is the most popular social media platform in China used by over one billion people (i.e., more than 70% of Chinese population) (22). The Wenjuanxing program has been widely used in epidemiological surveys (23, 24). Inclusion criteria included 1) adults aged 18 years or above; 2) frontline health professionals (i.e., doctors and nurses) working in isolation unit/hospitals, or fever clinics established for the COVID-19 outbreak in either outer emergency medical team from Liaoning in Wuhan or in Liaoning province; 3) ability to read Chinese and provide written informed consent. This study was approved by the clinical research ethics committee of the First Hospital of China Medical University.

### Measurements

Participant’s basic sociodemographic characteristics, such as gender, age, educational level, marital status, occupation, living circumstances, current smoking and drinking behaviors, current working status, previous working experience, and perceived family support, were collected using a data collection form designed for this study. Participant’s working status was assessed by four questions using a dichotomous response (“Yes/No”): 1) “Do you have direct contact with SARS-CoV-2 infected patient in daily clinical practice?”; 2) “Are you currently working in the COVID-19 outer emergency medical team in Wuhan, Hubei province?”; 3) “Are you familiar with the crisis response protocols and with relevant knowledge?”; and 4) “Have you ever attended any crisis response/rescue work previously?” Information on current drinking and smoking habits was solicited by the following questions: “Did you drink alcoholic beverage at least once per month (Yes/No)” (25), “Did you smoke at least one cigarette per day (Yes/No)” (26). Those who answered “Yes” to these questions were considered as current alcohol drinker or current smoker. ‘Perceived family support’ was measured by a single dichotomous question (Yes/No): “Do you think you had good familial support during the COVID-19 outbreak?”

The Chinese version of the PSQI is a 19-item instrument consisting of seven domains (subjective sleep quality, sleep latency, sleep duration, sleep efficiency, sleep disturbance, daytime dysfunction, and use of sleep medications). PSQI is a widely used self-administered questionnaire to assess sleep quality (27). The psychometric properties of the Chinese version of the PSQI was satisfactory, with the Cronbach’s alpha of 0.734 (28). The PSQI total score ranges from 0 to 21, with higher scores indicating poorer sleep quality. The cut-off value for poor sleep quality is 7 in Chinese populations (28).

### Data Analysis

Data analyses were conducted using SPSS Analytics software Version 21.0. Kolmogorov-Smirnov test was performed to test the normal distribution of continuous variables. Comparisons between good and poor sleep quality groups in terms of basic demographic and clinical characteristics were conducted using Chi-square test, and two independent samples t-test, as appropriate. Multivariate logistic regression analysis with the “enter” method (i.e., entering all the independent variables in the model simultaneously) was used to further identify significant independent demographic and clinical correlates associated with poor sleep quality. Poor sleep quality was the dependent variable, while all sociodemographic and clinical variables were entered as the independent variables. Significant level was set as P < 0.05 for all tests (two-sided).

## Results

A total of 1,931 health professionals participated in this study; of which, 355 (18.4%, 95%CI=16.6%–20.11%) reported poor sleep quality (PSQI total score of ≥7). **Table 1** shows the basic sociodemographic and clinical characteristics of participants by sleep quality. The mean PSQI total scores and component scores of the whole sample separated by good and poor sleep quality groups are shown in **Table 2**.

**Table 1:** Demographic characteristics of the study sample (N=1,931).

Variables	Whole sample (N=1,931)		Good sleep quality (N=1,576)		Poor sleep quality (N=355)		Statistics		
	n	%	n	%	n	%	*X^2^*	df	P
Male	88	4.6	73	4.6	15	4.2	0.110	1	0.740
High education (university and above)	1,862	96.4	1,524	96.7	338	95.2	1.865	1	0.172
Married	1,255	63.4	977	62.0	248	69.9	7.731	1	**0.005**
Nurses	1,614	83.6	1,300	82.5	314	88.5	7.509	1	**0.006**
Living with family	1,467	76.0	1,180	74.9	287	80.8	5.660	1	**0.017**
Current smoker	46	2.4	38	2.4	8	2.3	0.031	1	0.860
Current drinker	199	10.3	157	10.0	42	11.8	1.095	1	0.295
Working more than 5 years	1,438	74.5	1,147	72.8	291	82.0	12.878	1	**<0.001**
COVID-19 knowledge							0.305	2	0.859
Not familiar	325	16.8	267	16.9	58	16.3			
Familiar	921	47.7	747	47.4	174	49.0			
Very familiar	685	35.5	562	35.7	123	34.6			
Ever attending other crisis response	202	10.5	158	10.0	44	12.4	1.736	1	0.188
Familiar with crisis response knowledge	1,256	65.0	1,035	65.7	221	62.3	1.490	1	0.222
Working in outer response team in Wuhan	465	24.1	382	24.2	83	23.4	0.117	1	0.733
Contacting confirmed cases in daily work	249	12.9	201	12.8	48	13.5	0.152	1	0.697
Having family support	857	44.4	711	45.1	146	41.1	1.866	1	0.172
									
	Mean	SD	Mean	SD	Mean	SD	T	df	P
Age (years)	35.08	8.04	34.56	7.89	37.38	8.27	-6.034	1929	**<0.001**

Bolded values: < 0.05; M, mean; PSQI, Pittsburgh Sleep Quality Index; SD, standard deviation.

Good sleep quality was defined as Pittsburgh Sleep Quality Index (PSQI) < 7.

COVID-19, novel coronavirus disease.

**Table 2:** PSQI total and component scores in all participants.

	Total (N=1,931)		Good sleep quality (N=1,576)		Poor sleep quality (N=355)	
	M	SD	M	SD	M	SD
PSQI total score	4.61	3.36	3.39	2.17	10.00	2.20
Subjective sleep quality	0.96	0.85	0.71	0.68	2.05	0.67
Sleep latency	1.27	0.96	1.02	0.82	2.40	0.73
Sleep duration	0.21	0.64	0.10	0.40	0.69	1.11
Sleep efficiency	0.22	0.63	0.12	0.42	0.65	1.07
Sleep disturbance	0.76	0.63	0.61	0.52	1.46	0.58
Daytime dysfunction	1.03	0.97	0.78	0.81	2.12	0.84
Use of sleep medication	0.17	0.55	0.06	.027	0.63	1.02

M, mean; PSQI, Pittsburgh Sleep Quality Index; SD, standard deviation.

Univariate analyses revealed five correlates that were significantly associated with poor sleep quality (i.e., being nurses, older age, married, living with family, and > 5 years working experience), while the remaining were not associated with poor sleep quality. Multivariate logistic regression analysis revealed that nurses (OR=3.132, 95%CI=1.727–5.681, *P* < 0.001), older age (OR=1.043, 95%CI=1.026–1.061, *P* < 0.001), and health professionals who were working in outer emergency medical team in Hubei province (OR=1.755, 95%CI=1.029–3.064, *P*=0.039) were more likely to report poor sleep quality. Those who were familiar with crisis response protocols and with relevant knowledge were less likely to report poor sleep quality (OR=0.700, 95%CI=0.516–0.949, *P*=0.021) (**Table 3**).

**Table 3:** Independent correlates of poor sleep quality by multivariate logistic regression analysis.

Variables	Multivariate logistic regression analysis			
	OR	95% CI	P value
Age	1.043	1.026–1.061	**<0.001**
Female	0.711	0.372–1.360	0.302
High education (university and above)	0.751	0.421–1.340	0.333
Married	0.973	0.671–1.411	0.885
Nurses	3.132	1.727–5.681	**<0.001**
Living with family	1.020	0.639–1.628	0.933
Current smoker	0.746	0.318–1.753	0.502
Current drinker	1.162	0.786–1.717	0.451
Working more than 5 years	1.325	0.822–2.135	0.248
Not familiar with COVID-19 knowledge	ref	–	–
Familiar	0.879	0.541–1.426	0.601
Very familiar	0.798	0.473–1.348	0.399
Ever attending other crisis response	1.271	0.864–1.869	0.223
Familiar with crisis response knowledge	0.700	0.516–0.949	**0.021**
Working in outer response team in Wuhan	1.775	1.029–3.064	**0.039**
Contacting confirmed cases in daily work	0.901	0.501–1.618	0.726
Having family support	0.758	0.571–1.007	0.056

Bolded values: < 0.05; CI, confidence interval; COVID-19, novel coronavirus disease; OR, odds ratio.

## Discussion

This was the first study to examine sleep quality among frontline health professionals using the PSQI during the outbreak of the COVID-19 in areas less affected by COVID-19 in China. Using the cut-off value of 7, the prevalence of poor sleep quality was 18.4% (95%CI=16.6%–20.11%) among frontline health professionals in Liaoning province. This prevalence rate was lower than most of the previous findings in similar studies. For example, a recent cross-sectional study in China reported that 36.1% of frontline health professionals suffered from sleep disturbance using the Insomnia Severity Index (ISI) in early stage of the COVID-19 epidemic in China (i.e., late January, 2020) (7). A recent systematic review and meta-analysis found that the pooled prevalence of sleep disturbances among Chinese healthcare professionals was 39.2% (95%CI=36.0%–42.7%), using the PSQI (29). Machi *et al*. reported that the prevalence of poor sleep quality was 31.0% in US doctors working in emergency departments using the PSQI with the cut-off value of 6 (30), while Surani *et al*., reported that the prevalence of poor sleep quality was 36.8% in Pakistani physicians using the PSQI cut-off value of 5 (31). In contrast, the prevalence of poor sleep quality was 35.21% (95%CI=33.08%–37.35%) using the cut-off of 5, while the corresponding figure was 26.41% (95%CI=24.44%–28.38%) using the cut-off of 6 in this study.

The discrepancy in the prevalence of sleep quality in health professionals across studies could be partly explained by different population characteristics and the use of assessment tools. Since 25^th^ January, 2020, 30 provinces, municipalities, and autonomous regions covering over 1.3 billion Chinese population have initiated first-level responses to major public health emergencies. A range of measures, including establishment of emergency isolation infectious units and hospitals, have been urgently adopted (32, 33). However, compared to Hubei province, the epicenter of the COVID-19 in China, the disease epidemic was not as serious as in other areas of China. Liaoning province is a good example. Based on previous experience learned in the 2003 SARS epidemic that frontline health professionals were more likely to suffer from psychological problems (2), the authorities in Liaoning province have thus undertaken certain preventive interventions to relieve stress among frontline health professionals, such as timely provision of financial and material supports, mass education on pressure control, online psychological counseling service (e.g., 24-h hotlines), and on-site psychological guidance. These measures could reduce the risk of poor sleep quality (34).

As expected, older age was positively associated with poor sleep quality. Compared to their younger counterparts, older adults usually have more household responsibilities, and economic burdens (35, 36). Older adults are also prone to experience negative life events, such as divorce and bereavement, and suffer from physical discomforts and chronic physical diseases (37, 38), which could contribute to poor sleep quality (39). In this study, nurses were more likely to report poor sleep quality when compared with other health professionals (e.g., doctors and medical technicians). A vast majority of the nursing sample were females (95.4%). Some studies found that women were approximately 1.5 times more likely to report sleep problems than their male counterparts (30, 31, 40). Similar gender difference was also found in other neuropsychiatric diseases, such as headache, depression, and anxiety (40). In this light, generic factor could be one possible reason to explain the gender difference in sleep quality (41). It is also evident that anxiety and depression are more common in women, which could increase the risk of poor sleep quality (42–44). Besides, women tend to have greater bodily vigilance and awareness of physical symptoms than men. The societal norms and cultural context are more receptive for women to express their psychological distress and somatic symptoms (45), which may, perhaps, increase their likelihood of reporting poor sleep quality in survey studies.

Apart from female nurses, health professionals working in external emergency medical team in Hubei province, China were also more likely to experience poor sleep quality. In early February, 2020, emergency medical teams in Liaoning, China were urgently summoned to assist in Hubei province. Compared to those working in local isolation hospitals in Liaoning province, the external emergency medical teams need to adapt to unfamiliar living environment and work settings, and could experience heavy clinical workload, burnout, loneliness, homesickness, and fear of infection. All these bio-psycho-social factors could affect their sleep quality (46). Health professionals who were familiar with crisis response protocols/knowledge, however, were less likely to report poor sleep quality in this study. We speculate that receiving good training and learning relevant knowledge of crisis response for infectious diseases could be a protective factor and effectively reduce the extent of fear, anxiety, and uncertainty.

The strengths of this study include large sample size, and the use of standardized measurements. There are several methodological limitations that need to be acknowledged. First, this was a cross-sectional study, therefore, the causal relationships between demographic and clinical variables, and poor sleep quality could not be established. Second, most participants were female nurses, which could lead to potential selection bias. Third, sleep quality was assessed by only one self-administered instrument and thus, recall bias may exist. Finally, due to logistical reasons and risk of cross-infection, random sampling cannot be used in most studies on frontline health professionals during the COVID-19 outbreak. Thus, convenience sampling has been widely used (47, 48), which limits the generalizability of the findings. In addition, some variables associated with sleep quality, such as economic status, interpersonal relationship and psychiatric diagnoses (e.g., major depression and anxiety disorder), were not examined in this study.

In conclusion, it is encouraging to note that the prevalence of poor sleep quality was relatively low among frontline health professionals in Liaoning province during the COVID-19 epidemic. Nonetheless, considering the negative impact of poor sleep quality on health, wellbeing and daily clinical practice, regularly screening and timely treatments are warranted in frontline health professionals during the COVID-19 outbreak.

## Data Availability Statement

The raw data supporting the conclusions of this article will be made available by the authors, without undue reservation.

## Ethics Statement

The studies involving human participants were reviewed and approved by clinical research ethics committee of the First Hospital of China Medical University. The patients/participants provided their written informed consent to participate in this study.

## Author Contributions

Study design: YT and Y-TX. Data collection, analysis, and interpretation: YiZ, YY, TS, YS, YuZ, ZZ, YG, XL, YL, and GX. Drafting of the manuscript: YY, Y-TX, and YT. Critical revision of the manuscript: TC. Approval of the final version for publication: all co-authors.

## Funding

The study was supported by the University of Macau (MYRG2019-00066-FHS), and the National Key R&D Program of China (Grant #2018YFC1311600 and 2016YFC1306900).

## Conflict of Interest

The authors declare that the research was conducted in the absence of any commercial or financial relationships that could be construed as a potential conflict of interest.
